# Dragon kings of the deep sea: marine particles deviate markedly from the common number-size spectrum

**DOI:** 10.1038/srep22633

**Published:** 2016-03-04

**Authors:** Alexander B. Bochdansky, Melissa A. Clouse, Gerhard J. Herndl

**Affiliations:** 1 Ocean, Earth and Atmospheric Sciences, Old Dominion University, Norfolk, VA, USA; 2 Department of Limnology and Bio-Oceanography, Division Bio-Oceanography, University of Vienna, Althanstr. 14, 1090 Vienna, Austria; 3 Department of Biological Oceanography, Royal Netherlands Institute for Sea Research (NIOZ), 1790AB Den Burg, The Netherlands

## Abstract

Particles are the major vector for the transfer of carbon from the upper ocean to the deep sea. However, little is known about their abundance, composition and role at depths greater than 2000 m. We present the first number-size spectrum of bathy- and abyssopelagic particles to a depth of 5500 m based on surveys performed with a custom-made holographic microscope. The particle spectrum was unusual in that particles of several millimetres in length were almost 100 times more abundant than expected from the number spectrum of smaller particles, thereby meeting the definition of “dragon kings.” Marine snow particles overwhelmingly contributed to the total particle volume (95–98%). Approximately 1/3 of the particles in the dragon-king size domain contained large amounts of transparent exopolymers with little ballast, which likely either make them neutrally buoyant or cause them to sink slowly. Dragon-king particles thus provide large volumes of unique microenvironments that may help to explain discrepancies in deep-sea biogeochemical budgets.

Most of the sinking organic carbon in the ocean is remineralized by microbial activity and zooplankton feeding in the twilight zone (50–1000 m)^1^,^2^. However, the small percentage of particulate matter that escapes into the bathypelagic ocean is of interest because it represents a long-term loss of carbon from the surface layers when undergoing dissolution and transformation to refractory dissolved organic carbon, or when being buried in sediments. While most information about bathypelagic particles has come primarily from analyses of the contents of sediment traps^3^, a few surveys have explored particle numbers via optical means in their undisturbed state^4^,^5^,^6^, with the deepest bathypelagic number spectra reported to date from 1200–1400 m^7^ and 2500 m^8^,^9^. Optical surveys of the particle inventory are necessary because intact bathypelagic, flocculent marine snow is impossible to collect with traditional bottle samplers, and it has seldom been collected directly from submersibles^10^.

## Results and Discussion

**Figure 1:**
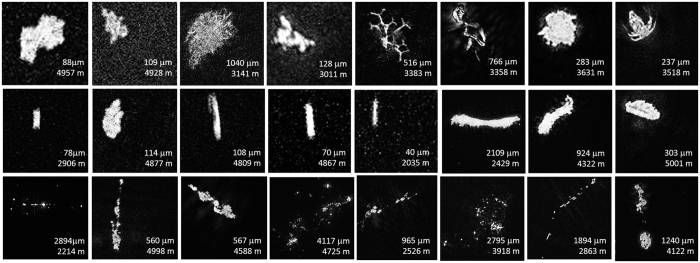
Examples of three categories of particles: marine snow (bottom row), faecal pellets (centre row) and “others” (top row). The “others” category includes all recognizable planktonic organisms (alive and carcasses) and optically dense debris that does not classify as marine snow or faecal pellets. For each image, the size (μm) and depth sampled (m) are given.

We deployed a custom-made digital inline holographic microscope (DIHM)^11^ at 16 stations in the subtropical and subarctic Atlantic and one station in the Arctic, with a maximum deployment depth of 5500 m (Supplementary Fig. 1). In contrast to lens-based systems, DIHM allows a focal depth of 7 cm, yielding relatively large volumes per image (1.8 mL) at high resolution. Three categories were considered: marine snow (which included amorphous aggregates – even those <500 μm – with and without ballast material such as faecal pellets and diatom frustules), individual faecal pellet-like particles (cylindrical and ovoid), and all “other” particles made of optically dense material (Fig. 1). The “other” category included single phytoplankton cells, optically dense debris, and heterotrophic plankton organisms (alive or as carcasses) (Fig. 1).

**Figure 2:**
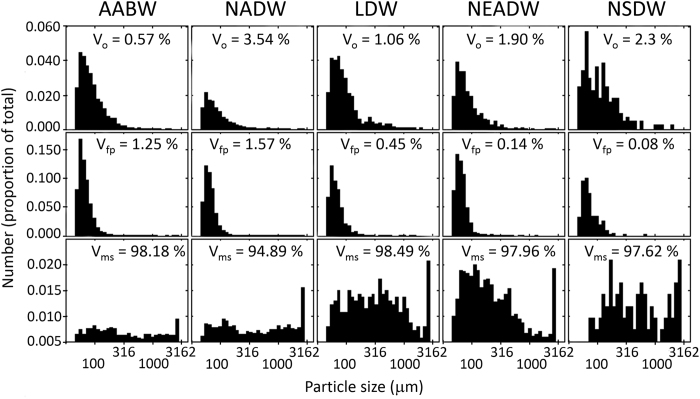
Size frequency distribution of three categories of particles in five different water masses: marine snow (bottom row), faecal pellets (centre row), and other particles including planktonic organisms (top row). AABW: Antarctic Bottom Water (n = 6,213), NADW: North Atlantic Deep Water (n = 13,824), LDW: Lower Deep Water (n = 1,408), NEADW: Northeast Atlantic Deep Water (including some mixed-in Labrador Sea Water, n = 3,610), NSDW: Norwegian Sea Deep Water (n = 440)^47^. The relative volume contribution for each of the particle types is given as a percentage: V_ms_ (marine snow) + V_fp_ (faecal pellets) + V_o_ (others) = 100%.

**Figure 3:**
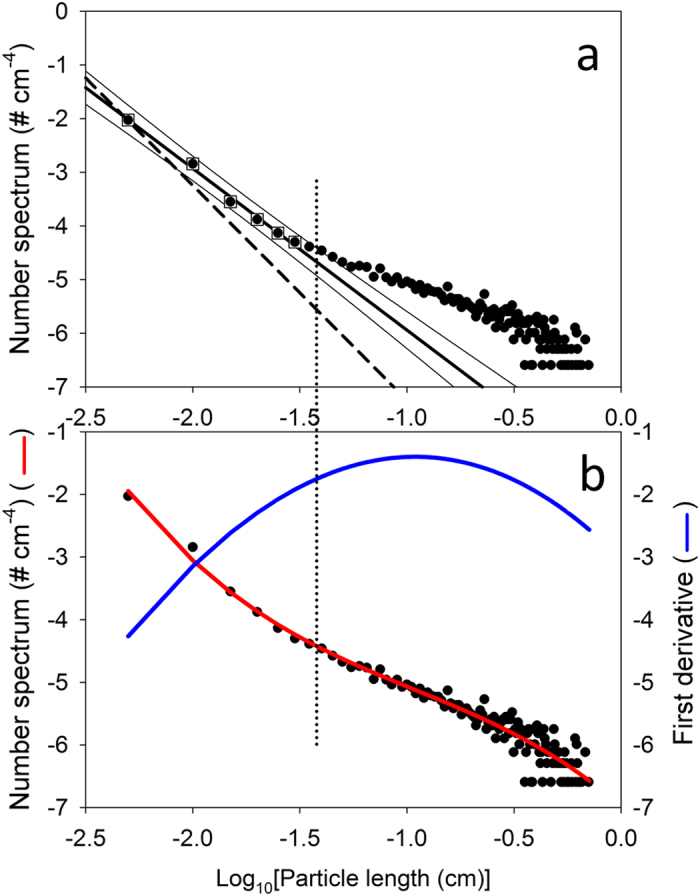
Particle number spectrum of deep sea (>1897 m) particles. (**a**) Linear regression model (thick solid line) based on smaller size classes (squares) is y = −8.95 − 3.01x, r^2^ = 0.994. Thin solid lines represent 95% prediction limits. The vertical dotted line indicates the lower limit of the dragon-king size domain (379 μm) based on the departure of the number-size spectrum from the upper 95% prediction limit. The Junge spectrum line (slope = −4) is shown as a dashed line. (**b**) Third-order polynomial fit through the number spectrum (red; y = −0.5532x^3^ − 1.6123x^2^ − 2.9697x −6.9761) and its first derivative (blue).

The frequency distribution of faecal pellets and other particles showed a typical distribution with a higher frequency of smaller particles (Fig. 2). However, the frequency of marine snow particles was more evenly spread, with a relatively high abundance of large particles in this group (Fig. 2). Consequently, the volume contribution of particles in the marine snow category (V_ms_) dominated the total volume of particles, with values ranging from 95% in North Atlantic Deep Water (NADW) to 98% in Lower Deep Water (LDW) (Fig. 2). The relative frequency of marine snow particles was higher in LDW, Northeast Atlantic Deep Water (NEADW), and Norwegian Sea Deep Water (NSDW) than in NADW and Antarctic Bottom Water (AABW). The number spectrum of all particles combined showed considerable deviation from a straight line (Fig. 3a) and was better described by a third-order polynomial (Fig. 3b). While the number spectrum from 50 μm to 300 μm was fit well by a linear regression with a slope of −3, it deviated sharply from the predicted relationship at larger sizes (Fig. 3a). The first derivative of the polynomial peaked at a particle size slightly larger than 1 mm, with a tangential slope value of −1.5 (Fig. 3b). The number spectrum returned to steeper slope values at particle sizes of several millimetres (Fig. 3b). Particles larger than 379 μm can be considered to be “dragon kings”^12^, i.e., events or phenomena to which usual power laws or abundance spectra do not apply and whose frequency of occurrence cannot be inferred from the distribution of more frequent events. Here, we define the dragon-king domain as the departure of the number spectrum from the 95% prediction interval of the linear regression through smaller size ranges, which occurred at a size of 379 μm (Fig. 3a). The definition is, therefore, not based on an arbitrary size cutoff but on the numerical dominance of a particle size range over others. This lower size threshold may be different for other samples and environments. In contrast, the definition of “marine snow” is arbitrary (e.g., particles >500 μm), and particles of this size range may not be considered dragon kings if their number spectrum falls within that predicted from smaller size classes. The consequence of the deviation from the number spectrum observed here is considerable; for instance, particles 400 μm in size were approximately twice as abundant as predicted from the regression with a slope of −3. Particles with sizes of 1 mm, 3.5 mm, and 8 mm (the largest observed size here) were 10, 44, and 90 times more abundant, respectively, than expected from a power law with a slope of −3 (Fig. 3).

The deviation is even more pronounced when contrasted with the commonly used benchmark slope of −4 (i.e., the Junge power law, dashed line in Fig. 3a)^13^,^14^,^15^. The Junge slope indicates that particle volumes are equal for equal logarithmic size intervals. This number-size distribution was originally assumed to hold true for the bathypelagic environment and was subsequently upheld by Coulter Counter measurements^14^. However, the upper limit of Coulter Counter measurements of 100 μm is below the point at which our data significantly diverged from a straight line (i.e., exceeded the 95% prediction interval). The Coulter Counter also creates artefacts because of aperture shear disaggregation^14^. For the smaller particle size range (50–100 μm), the slope in our study was shallower than that predicted by the Junge spectral slope and was closer to the slope of −3 previously reported for the surface ocean across many size ranges and instruments^16^. Particle spectra of marine systems are usually fit with one or several straight regression lines (on log-transformed values) with slopes ranging from −2 to −6^7^,^13^,^15^, and overall, the individual deviations level out to straight spectra^13^,^16^. Local deviations from linearity over distinct size classes in the upper ocean have been attributed to processes such as cell growth, faecal pellet production, coagulation driven by diel cycles in turbulence, disaggregation, and ingestion by zooplankton^13^,^17^. The differential settling of larger particles over smaller ones certainly contributes to the increased relative abundance of larger particles in the deep sea. Some previously reported particle spectra showed similar deviations from straight slopes^7^,^8^,^9^; thus, the deviation we describe here may not be restricted to depths >2000 m. However, the flattening of particle spectra at the larger particle size range^13^,^15^ could also be the result of undersampling and a truncation effect caused by bins with zero values^16^. In contrast, the number spectrum reported here returned to steeper slope values at particle sizes >1 mm, which means that undersampling did not bias the deviations from the initial slope at 379 μm. It is extremely important for the spectral analysis and the identification of dragon kings to ensure that particles at both ends of the size spectrum are sampled with 100% efficiency. Otherwise, the number spectrum may be artificially curved. In our analysis, we had to exclude a large number of particles from the analysis at the lower size range (<50 μm) because they could not be sampled with 100% efficiency (see Methods). Particles that were identifiable as plankton organisms were a small fraction in the dragon-king size domain (i.e., 13.1%), and most of those were diatoms (82% of those identified as organisms or parts of organisms). Thus, amorphous marine snow aggregates were primarily responsible for the nonlinearity in the spectrum observed here.

**Figure 4:**
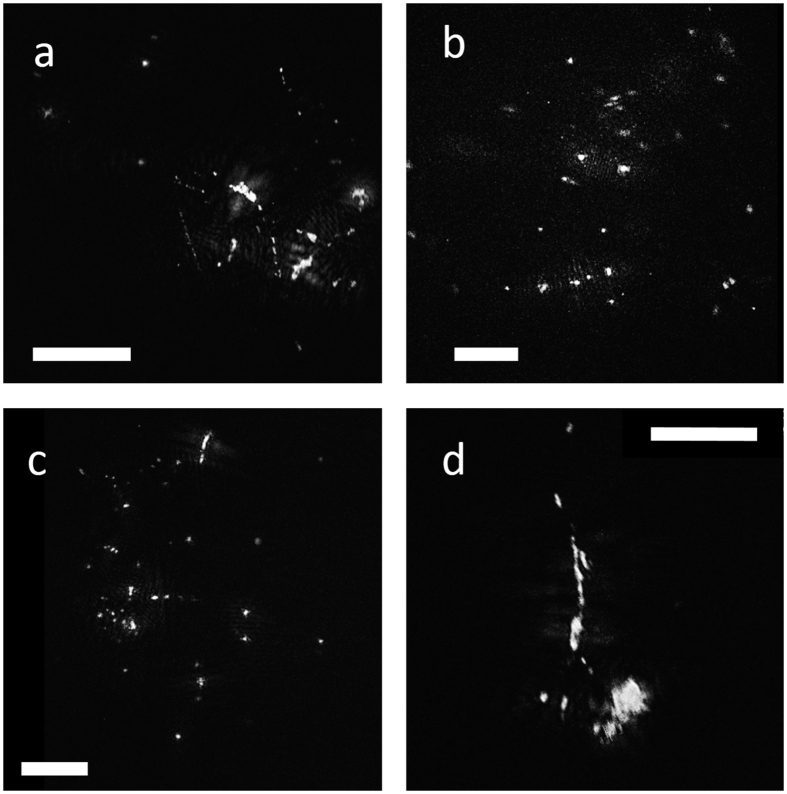
Examples of dragon-king particles with little apparent ballast (**a–c**) and a ballasted stringer-type particle (**d**). Particles are held together by a large amount of transparent exopolymers. White scale bars = 1 mm.

**Figure 5:**
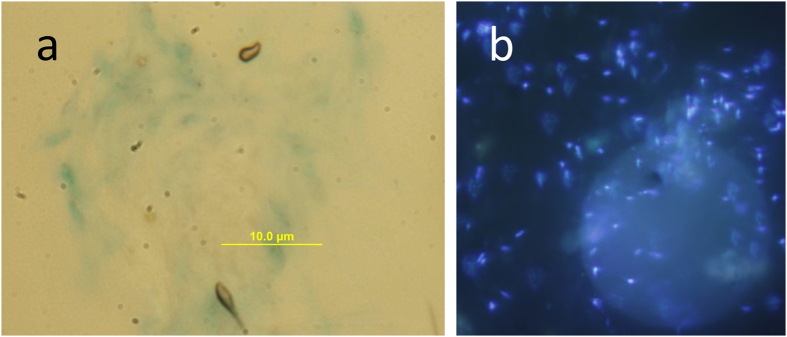
Example of Alcian Blue-stained TEP matrix that holds larger particles together (**a**), and a particle lying across a 30-μm pore of a membrane filter as observed under the epifluorescence microscope (**b**). The transparent exopolymer matrix is heavily colonized by prokaryotes (blue) visualized with 4′,6-diamidino-2-phenylindole (DAPI). The transparent matrix is too thick to show all prokaryotes in focus simultaneously.

Particles collected in polyacrylamide gel traps allow a direct comparison with those captured by *in situ* optical instruments^18^. Gel traps are mostly dominated by “faecal aggregates” (ballasted by denser material), cylindrical and ovoid faecal pellets, and optically dense phytoplankton aggregates^19^,^20^,^21^. In some instances, phytodetrital aggregates dominate the flux numerically but not in terms of carbon because the density of faecal pellets is higher^22^. Amorphous marine particles with low-density material (“fluff aggregates^20^”) are rare in polyacrylamide gel traps, for instance contributing only 0 to 4% numerically and even less volumetrically^20^. In contrast, in our analysis, dragon-king particles contained large amounts of transparent exopolymers (Figs 4 and 5). Overall, 32 ± 14.5% (n = 17 stations) of particles >379 μm resembled low-density, porous, and amorphous aggregates. This transparent material is well known to be a major contributor to the formation and matrix of marine snow; however, it is invisible unless stained by Alcian Blue (Fig. 5) or Coomassie Brilliant Blue^23^. It is less dense than seawater and thus increases the buoyancy of particles^24^. Generally, there is only a loose relationship between particle size and sinking velocity because predictions based on the Navier-Stokes law usually underestimate the sinking velocities of small particles and overestimate those of large amorphous aggregates^25^,^26^,^27^. Both the large amount of optically transparent material in the particle matrix and the almost complete absence of these particles in gel traps^20^ suggest that they are either neutrally buoyant or sink only slowly. Slowly sinking, horizontally transported particles have previously been suspected to be a major source of error in budget calculations for the deep sea, but little information exists because they are severely undersampled by sediment traps^28^.

We can only speculate on the origin of the dragon-king particles. They do not appear to be made of discarded appendicularian houses^29^ because they lack the typical dense cluster of small particles in the region of the food-concentrating filter. Their dominance could be the result of differential settlement to deeper layers if most of the smaller particles are solubilized before they reach the bathypelagic layers. Small particles are also not produced at the same rate as in surface environments because of the much lower production rates of microbes at depth^30^. Coagulation by shear and differential settlement with the contribution of prokaryotes^31^ may also slowly build larger particles from smaller ones at depth, and it has been suggested that the deep sea environment is conducive to the self-assembly of gels^32^. Their accumulation may be facilitated by the fact that organisms known to significantly consume and fragment similar-sized particles in the mesopelagic layer^1^,^33^ are generally absent from bathy- and abyssopelagic environments^34^. Particles identifiable as plankton or their parts only represented 0.1% of the total number of particles analysed here.

While dragon-king particles may not contribute substantially to the vertical flux of organic material, they likely play a major role in deep sea ecosystems as resource-rich habitats for microbes. We collected particles onto 30-μm membrane filters using gentle gravity filtration directly from Niskin bottles and found that the transparent matrix was heavily colonized by prokaryotes and protists (Fig. 5). This result is not unexpected because gels have an increased concentration of organic matter, approximately 1000 times greater than the surrounding seawater^35^. Direct collection of particles >3 mm in the mesopelagic by submersibles has revealed prokaryotes to be 4 orders of magnitude more concentrated than in the ambient water (i.e., approx. 10^8^ *vs*. 10^4^ mL^−1^, Fig. 2 therein^36^). This enrichment most likely holds true for bathypelagic particles as well, given that ambient prokaryote concentrations are approximately 100 times lower than those at the surface where enrichments on particles typically range from 100 to 1000 x^37^. It has been suggested that the solubilization of particles is faster than their remineralization^38^, resulting in an abundance of dissolved matter in the pore water of marine snow. This enrichment in dissolved organic material is important because thresholds for nutrient uptake in particle pore water and their plumes most likely exceed the minimum concentration required for the growth of deep-sea prokaryotes^39^. Chemical microenvironments, such as those with low oxygen, can persist in particles, facilitating processes such as denitrification^40^, and methane and ammonium production in the water column^41^,^42^. Recent accounts have demonstrated the presence of quorum sensing on particles^43^, and models suggest that diffusion and remineralization are not only influenced by the relative abundance of microbes but also by their location on particles^44^. All these factors combined may lead to the dominant contribution of particle-associated microbes to the overall metabolism of the deep sea^45^. These findings also challenge traditional bottle incubation methods. If microbial processes are tied to the integrity of particles that are too fragile to be collected but which contain unique communities and microenvironments, the typical bulk collection and incubation of water samples is inadequate to produce accurate estimates of metabolic rates for the ocean’s interior. Dragon-king particles may have been overlooked with traditional sampling methods such as sediment traps, but the dominance of this size class suggests that particle-associated small-scale heterogeneity needs to be recognized in an environment that represents the largest oceanic subsystem in terms of volume.

## Methods

### Digital Inline Holographic Microscopy

Details of the custom-made digital inline holographic microscope for the deep sea have been published elsewhere^11^. The path length of the laser (640 nm) through the water was 7 cm. The DIHM was mounted on the lowest point of the CTD rosette frame to leave an unobstructed path for the water to pass between the point source and the camera. Only down casts were used for analysis to avoid imaging particles that were fragmented by the instrument cradle and wire. Vertical speeds through the water ranged from 1 to 1.5 m sec^−1^. Seven to twelve 4-megapixel images were recorded per second. While the maximum resolution per image is approximately 5 μm, only particles larger than 50 μm can be reliably enumerated in the entire image volume^11^. Each image represents a volume of 1.8 mL, and all particles can be brought into focus within this volume. A total of 46,275 images were reconstructed using Octopus reconstruction software by 4-Deep (formerly Resolution Optics, Halifax, Canada) using the Kirchhoff-Helmholtz transform^46^. The maximum length of the particle was measured manually using the built-in measuring tool, and equivalent spherical volumes were calculated from the maximum lengths. Supplementary Fig. 1 and Supplementary Table 1 show the stations sampled, the depth intervals, and the water masses in which particles were measured and characterized. The depths were chosen to target specific water masses^47^. The particle size - number spectrum (differential size spectrum) was calculated as *n(s)* = −*dN/ds*, where *n* is the particle size spectrum, *N* is the cumulative particle size distribution, and *s* is the maximum linear dimension of the particle^16^. Of 46,275 images, only particles ≥50 μm were considered in the analysis of the number spectrum (n = 20,552) because they can be reliably enumerated in the image beam^11^. This avoided a bias in the lower range of the particle size spectrum^16^. In Fig. 3, this particle size spectrum was compared to the Junge slope of −4 and a slope of −3, the most frequently reported slopes in studies of marine surface environments^13^,^16^.

### DIHM analysis of the dragon-king size domain

To calculate the percentage value of amorphous aggregates >379 μm that contained a large amount of optically transparent material, a second survey of images was performed. Instead of reconstructing all particles within a depth range, the image sequence was stopped only when a large particle was encountered in the raw images. In the expanding beam configuration of the holography applied here, all large particles can be reliably captured in this fashion. Some particles smaller than the threshold criterion that were also captured by this method were not included in the percentage calculation.

### Gravity filtration

The images in Fig. 5 are based on gentle gravity filtration directly from a 25-L Niskin bottle onto a Millipore polycarbonate filter (30 μm pore size, 25 mm diameter). The maximum flow rate was kept to 100 mL min^−1^ with a flow restrictor placed inline after the filter cartridge. The filter was subsequently fixed with 2% (fin. conc.) formaldehyde and stored at −80 °C. For the visualization of TEP, pie-shaped slices of 30-μm filters were placed on a 0.2-μm backing filter stained with Alcian Blue and mounted on Cyto-Clear slides^48^,^49^. The filter was then gently washed with ultrapure water. This procedure causes additional losses of particles from the filter and can thus only be used qualitatively. For visualization of prokaryotes in the gel matrix, the filter was first coated with agarose^50^ to avoid the detachment of particles and treated with 25 mM EDTA^51^ to make TEP permeable to the nucleic acid stain. The filter was then mounted on a slide with an antifadent solution containing 4′,6-diamidino-2-phenylindole (Vectashield with DAPI).

## Additional Information

**How to cite this article**: Bochdansky, A. B. *et al*. Dragon kings of the deep sea: marine particles deviate markedly from the common number-size spectrum. *Sci. Rep.* **6**, 22633; doi: 10.1038/srep22633 (2016).

## Supplementary Material

Supplementary Information
