# Supplementary material for: Dragon kings of the deep sea: marine particles deviate markedly from the common number-size spectrum
Source: Sci Rep. 2016 Mar 4;6:22633. doi: 10.1038/srep22633 (PMC4778057; doi:10.1038/srep22633)
Supplement: Supplementary Information [file srep22633-s1.doc]

**Supplementary Data**

**Dragon kings of the deep sea: major deviation of particles from the common number - size spectrum.**

Alexander B. Bochdansky^1*^, Melissa A. Clouse^1^, Gerhard J. Herndl^2^

^1^Ocean, Earth and Atmospheric Sciences, Old Dominion University, Norfolk, VA, USA

^2^Department of Limnology and Bio-Oceanography, Division Bio-Oceanography, University of Vienna, Althanstr. 14, 1090 Vienna, Austria and Department of Biological Oceanography, Royal Netherlands Institute for Sea Research (NIOZ), 1790AB Den Burg, The Netherlands

* abochdan@odu.edu

Figure 1. Map of stations at which the DIHM was deployed.


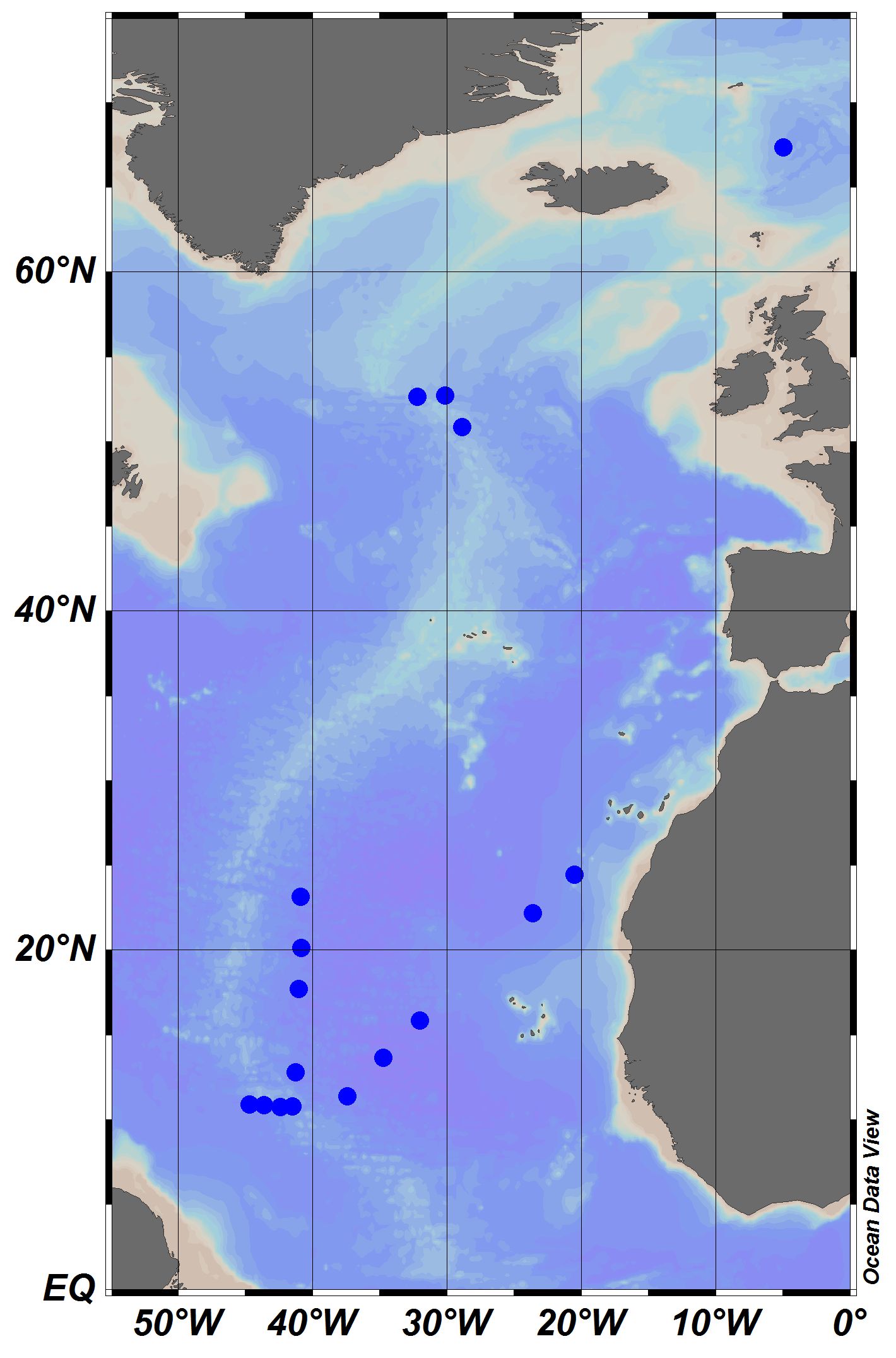


Map was created with Ocean Data View (Schlitzer, R., Ocean Data View, odv.awi.de, 2015)

Table 1. Cruise name (A4 = Archimedes 4, M2 = Medea 2), station number, dates, and positions for DIHM deployments. The depth range of the particle analysis targeted representative sections of water masses^45^: NADW = North Atlantic Deep Water; AABW = Antarctic Bottom Water; LSW = Labrador Sea Water; NEADW = Northeast Atlantic Deep Water; LDW = Lower Deep Water; NSDW = Norwegian Sea Deep Water;

| Cruise | Station | Date | Longitude (degree E) | Latitude (degree N) | Depth range of analysis  (m) | Water mass  (van Aken 2000) |
| --- | --- | --- | --- | --- | --- | --- |
| A4 | 2 | 11Oct2010 | -20.49 | 24.45 | 2870-3070 | NADW |
| A4 | 2 | 11Oct2010 | -20.49 | 24.45 | 3703-3903 | AABW |
| A4 | 3 | 12Oct2010 | -23.58 | 22.17 | 1980-2180 | NADW |
| A4 | 3 | 12Oct2010 | -23.58 | 22.17 | 2900-3100 | NADW |
| A4 | 3 | 12Oct2010 | -23.58 | 22.17 | 4600-4914 | AABW |
| A4 | 6 | 15Oct2010 | -32.03 | 15.86 | 3093-3293 | NADW |
| A4 | 6 | 15Oct2010 | -32.03 | 15.86 | 5100-5354 | AABW |
| A4 | 7 | 16Oct2010 | -34.73 | 13.64 | 1900-2101 | NADW |
| A4 | 7 | 16Oct2010 | -34.73 | 13.64 | 2465-2564 | AABW |
| A4 | 7 | 16Oct2010 | -34.73 | 13.64 | 3262-3356 | NADW |
| A4 | 7 | 16Oct2010 | -34.73 | 13.64 | 4461-4618 | AABW |
| A4 | 7 | 16Oct2010 | -34.73 | 13.64 | 5000-5200 | AABW |
| A4 | 7 | 16Oct2010 | -34.73 | 13.64 | 5305-5505 | AABW |
| A4 | 8 | 17Oct2010 | -37.43 | 11.38 | 1897-2097 | NADW |
| A4 | 8 | 17Oct2010 | -37.43 | 11.38 | 3318-3546 | NADW |
| A4 | 8 | 17Oct2010 | -37.43 | 11.38 | 5004-5204 | AABW |
| A4 | 11 | 20Oct2010 | -41.48 | 10.77 | 1938-2138 | NADW |
| A4 | 11 | 20Oct2010 | -41.48 | 10.77 | 3240-3440 | AABW |
| A4 | 11 | 20Oct2010 | -41.48 | 10.77 | 4800-5000 | AABW |
| A4 | 12 | 21Oct2010 | -42.4 | 10.76 | 3276-3476 | NADW |
| A4 | 12 | 21Oct2010 | -42.4 | 10.76 | 4800-5000 | AABW |
| A4 | 13 | 22Oct2010 | -43.59 | 10.85 | 1897-2097 | NADW |
| A4 | 13 | 22Oct2010 | -43.59 | 10.85 | 4825-5025 | AABW |
| A4 | 14 | 23Oct2010 | -44.67 | 10.91 | 3300-3500 | NADW |
| A4 | 14 | 23Oct2010 | -44.67 | 10.91 | 3506-3519 | NADW |
| A4 | 14 | 23Oct2010 | -44.67 | 10.91 | 4800-5000 | AABW |
| A4 | 14 | 23Oct2010 | -44.67 | 10.91 | 5000-5002 | AABW |
| A4 | 15 | 24Oct2010 | -41.28 | 12.78 | 2295-2495 | NADW |
| A4 | 15 | 24Oct2010 | -41.28 | 12.78 | 3394-3594 | NADW |
| A4 | 15 | 24Oct2010 | -41.28 | 12.78 | 4601-4727 | AABW |
| A4 | 17 | 26Oct2010 | -41.05 | 17.71 | 2684-2884 | NADW |
| A4 | 17 | 26Oct2010 | -41.05 | 17.71 | 3245-3456 | NADW |
| A4 | 17 | 26Oct2010 | -41.05 | 17.71 | 4485-4509 | AABW |
| A4 | 18 | 27Oct2010 | -40.84 | 20.12 | 2646-2846 | NADW |
| A4 | 18 | 27Oct2010 | -40.84 | 20.12 | 3147-3347 | NADW |
| A4 | 18 | 27Oct2010 | -40.84 | 20.12 | 4800-5000 | AABW |
| A4 | 19 | 28Oct2010 | -40.89 | 23.13 | 2600-2900 | NADW |
| A4 | 19 | 28Oct2010 | -40.89 | 23.13 | 3400-3600 | NADW |
| A4 | 19 | 28Oct2010 | -40.89 | 23.13 | 4000-4396 | AABW |
| A4 | 19 | 28Oct2010 | -40.89 | 23.13 | 4900-5021 | AABW |
| M2 | 7 | 2Jul2012 | 331.14 | 50.86 | 1949-2049 | LSW |
| M2 | 7 | 2Jul2012 | 331.14 | 50.86 | 2700-2800 | NEADW / LSW |
| M2 | 7 | 2Jul2012 | 331.14 | 50.86 | 2899-3099 | NEADW |
| M2 | 15 | 10Jul2012 | 327.80 | 52.64 | 2000-2106 | LSW |
| M2 | 15 | 10Jul2012 | 327.80 | 52.64 | 2450-2595 | NEADW/LSW |
| M2 | 15 | 10Jul2012 | 327.80 | 52.64 | 2950-3060 | NEADW |
| M2 | 15 | 10Jul2012 | 327.80 | 52.64 | 3150-3250 | LDW |
| M2 | 16 | 11Jul2012 | 329.86 | 52.73 | 1900-2100 | LSW |
| M2 | 16 | 11Jul2012 | 329.86 | 52.73 | 2650-2850 | NEADW |
| M2 | 16 | 11Jul2012 | 329.86 | 52.73 | 3000-3200 | LDW |
| M2 | 24 | 19Jul2012 | 355.06 | 67.35 | 1899-2099 | NSDW |
| M2 | 24 | 19Jul2012 | 355.06 | 67.35 | 2500-2700 | NSDW |
| M2 | 24 | 19Jul2012 | 355.06 | 67.35 | 3100-3300 | NSDW |
